# Improving Texture and Protein Content in 3D-Printed Plant-Based Foods for Dysphagia: A Study of Pea-Protein and Curcumin-Enriched Oleogel Formulations

**DOI:** 10.3390/foods15071125

**Published:** 2026-03-25

**Authors:** Heremans Camille, Baugier Benjamin, De Rijdt Mathieu, Bradfer Roxane, Potvin Nelly, Ayadi Mohamed, Haubruge Eric, Goffin Dorothée

**Affiliations:** 1 Laboratory of Gastronomic Sciences, Gembloux Agro-Bio Tech, Université de Liège, 5030 Gembloux, Belgium; benjamin.baugier@student.uliege.be (B.B.); mderijdt@uliege.be (D.R.M.); e.haubruge@uliege.be (H.E.); 2 Qualité et Sécurité des Produits Agro-Alimentaires, Gembloux Agro-Bio Tech, Université de Liège, 5030 Gembloux, Belgium

**Keywords:** texture-modified foods, additive manufacturing, plant proteins, IDDSI, food texture, dysphagia

## Abstract

Texture-modified foods (TMFs) are essential for individuals with dysphagia, yet conventional formulations often lack structural consistency, nutritional density, and sensory appeal. Three-dimensional (3D) food printing offers new opportunities to tailor texture and composition. This study developed 3D-printed TMFs based on a lentil-carrot matrix and formulated with pea protein isolate (PPI), a curcumin-enriched oleogel (O), or their combination (PPI–O), and compared them with a commercial dysphagia thickener reference. Printability was assessed through extrusion force measurements and dimensional deviation analysis. Texture profile analysis (TPA), International Dysphagia Diet Standardisation Initiative (IDDSI) tests, moisture and protein content determination, color measurements, and preliminary sensory evaluation were conducted. PPI-containing formulations required higher extrusion forces but showed improved dimensional stability, hardness, cohesiveness, and gumminess compared with the oleogel-only sample, likely due to the formation of a stronger protein network. In contrast, the oleogel-only formulation exhibited lower mechanical resistance and a more pronounced melting perception, reflecting the lubricating effect of the lipid-based matrix. Protein content significantly increased with PPI incorporation, and curcumin-enriched oleogel also markedly influenced color parameters. All samples were classified as compatible with IDDSI Level 5. The hybrid PPI–O formulation provided a balanced combination of printability, structural fidelity, enhanced protein content, and suitable textural properties. These findings suggest that extrusion-based 3D printing may represent a promising approach for designing plant-based TMFs for dysphagia-oriented foods.

## 1. Introduction

Dysphagia is defined as an impaired ability to transport food or liquids from the oral cavity to the stomach in a safe and effective manner [1]. This condition is highly prevalent among older adults and frequently affects individuals with neurological disorders, head and neck cancers, or post-stroke complications [2,3]. With global life expectancy continuing to rise, the number of people living with dysphagia is expected to increase accordingly. Beyond swallowing impairment itself, dysphagia is associated with severe consequences such as malnutrition, dehydration, weight loss and aspiration pneumonia. Consequently, ensuring safe and adequate nutrition for dysphagic individuals remains a critical public health challenge [1,4,5].

To mitigate these risks, texture-modified foods (TMFs) are widely prescribed, as they help reduce aspiration events and improve swallowing comfort [6,7]. However, despite their essential clinical role, TMFs often suffer from poor sensory and visual appeal [5]. Many patients perceive them as unappetizing, which decreases adherence and may further worsen malnutrition. Ensuring that TMFs are both safe to swallow and enjoyable to eat is therefore crucial [3]. Recent strategies have incorporated protein fortification to counteract age-related sarcopenia and insufficient dietary intake, yet achieving a product which is simultaneously palatable, protein-dense and visually appealing remains challenging [8,9,10].

The International Dysphagia Diet Standardisation Initiative (IDDSI) provides a standardized classification framework for liquids and foods based on their texture and flow characteristics [2]. This system is increasingly adopted worldwide to enhance safety, consistency and reproducibility in dysphagia management. While several national and regional dysphagia diet frameworks, such as the National Dysphagia Diet (NDD) developed by the American Dietetic Association, were historically implemented, they have largely been superseded by harmonized international approaches [11]. For solid and semi-solid foods, IDDSI levels range from liquidized (Level 3) to regular textures (Level 7), with specific tests such as the spoon tilt, fork drip and fork pressure methods guiding classification [11,12]. Although IDDSI has significantly improved standardization, it does not address the persistent challenge of producing TMFs that are not only safe and nutritionally adequate but also appealing and desirable for patients. Moreover, this framework does not constitute clinical validation of swallowing safety or patient-specific suitability, which require assessment in the target population.

In parallel, three-dimensional (3D) food printing has emerged as a promising approach capable of addressing several limitations associated with conventional TMFs [13,14]. This additive manufacturing technology is a layer-by-layer process allowing precise control over structural design, textural attributes and visual presentation, enabling manipulation of texture to enhance safety and swallowing comfort [13,15,16,17]. By allowing fine manipulation of texture to improve swallowing safety while enhancing visual appeal, 3D printing has the potential to substantially increase patient acceptance and adherence [16]. Nevertheless, several technical challenges remain, particularly those related to extrusion behavior, formulation-dependent rheological properties, ingredient interactions, and post-deposition structural stability, all of which critically influence shape fidelity and overall printability [17].

Hydrocolloids play a central role in overcoming these technical limitations. Xanthan gum, in particular, is widely used due to its pronounced shear-thinning behavior: it decreases viscosity during extrusion, facilitating smooth flow through the nozzle and rapidly recovers after deposition, thereby ensuring dimensional stability of the printed structure [18,19]. As a result, xanthan gum is a key component for achieving both printability and post-print structural integrity.

Beyond hydrocolloids, growing attention has been directed towards functional food ingredients such as pea protein isolate (PPI) and oleogel (O). PPI is a high-quality plant-protein source that provides essential amino acids, thereby enhancing the protein composition of TMFs. In addition, PPI contributes to structural reinforcement through its ability to form a cohesive protein network [17,20,21]. Its plant-based origin and low-allergen potential further make it particularly suitable for elderly and dysphagic populations [22]. Oleogels, obtained by structuring edible oils into semi-solid matrices, offer tunable rheological and textural properties representing promising alternatives to conventional fats. Their semi-solid structure and shear-dependent flow behavior can also support extrusion-based 3D printing by facilitating material flow through the nozzle while helping the deposited layers maintain their shape. They can improve mouthfeel while enabling fat reduction or fat replacement strategies. Moreover, due to their lipophilic nature, oleogels are effective carriers for poorly water-soluble bioactive compounds [23,24,25,26]. Curcumin, a polyphenolic compound widely recognized for its antioxidant and anti-inflammatory properties, is one such compound that benefits from oleogel encapsulation, which improves its stability and bioavailability by protecting it from degradation [23,27].

Combining PPI with curcumin-enriched oleogels represents a promising strategy for the development of compositionally enhanced, plant-based TMFs with improved structural, functional and sensory properties. This approach may contribute to addressing key challenges in the development of 3D-printed foods for dysphagia, including achieving adequate texture and printability, protein enrichment, and the incorporation of functional ingredients. To the best of our knowledge, while oleogels have previously been explored in 3D food printing and protein enrichment has been investigated to improve dysphagia-oriented foods, the combined use of PPI and oleogel within a single 3D-printed TMF formulation has not yet been reported. However, in the present study, curcumin is used primarily as a model lipophilic compound to investigate its incorporation within oleogel-based printed matrices, and no claims regarding functional delivery or bioavailability are made.

The purpose of this study is to develop a lentil-carrot-based formulation suitable for 3D printing and intended for dysphagic patients using PPI and a curcumin-enriched oleogel. The techno-functional properties of the developed formulations are compared with those of a commercial dysphagia thickener. The formulations are evaluated through assessments of printability, IDDSI classification, texture profile analysis (TPA), moisture content, protein content, colorimetric properties and a preliminary sensory evaluation. Together, these findings support the development of next-generation dysphagia-oriented foods enabled by advanced 3D printing technologies.

## 2. Materials and Methods

### 2.1. Preparation of 3D Printing Materials

A plant-based matrix was prepared using a 6:1 green lentil (*Lens culinaris *Medik., CRAW, Gembloux, Belgium) and carrot formulation (Delhaize, Brussels, Belgium) (*w*/*w*). The lentils were soaked in water (1:3 *w*/*v*) overnight (≈12 h) at room temperature and subsequently steamed for 45 min at atmospheric pressure (≈100 °C) using a conventional saucepan. Peeled and chopped carrots were steamed for 30 min under identical conditions. Green lentils and carrots were then drained and blended (Vitamix, Cleveland, OH, USA) to obtain a homogeneous purée, which served as the base for all samples. The reference sample consists of this lentil-carrot matrix supplemented with 4% (*w*/*w*) of added commercial instant thickener (Thick & Easy Clear, Fresenius, Bad Homburg, Germany), purchased at a pharmacy and intended for thickening foods and beverages. This functional powder contains dextrin-maltose, xanthan gum, carrageenan and erythritol, and the amount to be mixed into meals is prescribed by healthcare professionals based on the stage of the swallowing disorder. The 4% concentration was chosen as a mid-range value within the manufacturer’s recommended dosage guidelines.

Regarding the three experimental samples, 0.4% XG (*w*/*w*) (Texturas Ferran Adria, Barcelona, Spain) was added as a thickening agent to the lentil–carrot base. Depending on the experimental condition, 4.5% pea protein isolate (*w*/*w*) (PISANE™ ES pea protein, Cosucra, Warcoing, Belgium) was incorporated. In addition, 8% of oleogel was either included or omitted. The levels of xanthan gum, pea protein isolate, and oleogel were determined based on preliminary optimization tests, with the tested concentration ranges initially defined according to values reported in the literature [7,13,16,20,27]. The oleogel was prepared using 5% beeswax (*w*/*w*) (melting point: ~62–65 °C) (Dille & Kamille, Utrecht, The Netherlands) and heated rapeseed oil (Delhaize, Brussels, Belgium), with 7% curcumin (*w*/*w*) (Curcumin 95%, PIT & PIT, Ghent, Belgium) incorporated as a model bioactive compound. The oleogel was heated above the beeswax melting point to ensure complete dissolution, then mixed with the lentil–carrot base before solidifying to obtain a homogeneous formulation. All samples were prepared under identical processing conditions to allow direct comparison of their printability, stability, composition and texture. Ingredients and proportions of the different TMFs formulations are shown in Table 1.

### 2.2. 3D Printing Process

3D printing was performed using an extrusion-based food printer (Felix Food V1.5, Felix Printers, Aalst, Belgium). The printing workflow involved two stages: computer-aided design and material deposition. Three-dimensional models were created using Shapr3D software, version 26.40.10572.0 (Shapr3D Zrt., Budapest, Hungary) and exported as STL files. These files were converted into G-code using Simplify3D, version 5.1.2 (Simplify3D LLC, Cincinnati, OH, USA) for printer execution.

The formulations were extruded through a pneumatic syringe system controlled by a stepper-driven piston, enabling continuous and reproducible material flow [7]. For the printing experiments, the key parameters were configured as follows: syringe diameter, 30 mm; printing speed, 1800.0 mm/min (corresponding to the nozzle travel speed in the XY plane); nozzle diameter, 1.6 mm; layer height, 1.0 mm; and printing temperature, ambient temperature. Material deposition was controlled in real time using Simplify3D by applying an extrusion multiplier of 1.07, which was kept constant across all formulations to ensure consistent flow and filament continuity.

A standard cube model (25 × 25 × 25 mm) was printed for all formulations to allow consistent comparison of dimensional fidelity and techno-functional behavior. These dimensions were selected because they are rapid to print and enable reliable post-processing analyses. All analyses were conducted immediately after printing, with samples kept under ambient conditions and tested within 30 min of fabrication to minimize structural or moisture-related changes.

### 2.3. Extrusion Behavior

The extrusion behavior required during printing was evaluated using a texture analyzer (Lloyd TA1, Ametek, Berwyn, PA, USA) equipped with a 50 mm cylindrical probe. The formulation was loaded into the same syringe geometry as used during 3D printing, a 30 mm inner-diameter syringe fitted with a 1.6 mm diameter and 1 cm length nozzle, to ensure identical extrusion conditions. All tests were performed at ambient temperature. During the test, the plunger was displaced at a constant speed of 0.05 mm/s. A 1 N preload was applied at 120 mm/min to ensure consistent contact between the probe and the piston. The material was then extruded over a 30 mm compression distance, followed by a 60 s holding period.

From the force–displacement curves, two printability indicators were extracted: the steady-state force (N), which is the average force during the plateau phase, and the compression energy (N·mm), referring to the mechanical work required for extrusion. These indicators correspond to commonly reported descriptors of extrudability and printability for food pastes and semi-solid formulations [28].

To relate the measured forces to the internal hydrodynamic conditions within the syringe, the extrusion pressure (P) was computed by normalizing the steady-state force by the cross-sectional area of the syringe barrel:

$$P=F/A_{\mathrm{syringe}}\;\mathrm{with}\;A_{\mathrm{syringe}}=\pi {\left(\frac{D}{2}\right)}^{2},\mathrm{where}\;D=30\;\mathrm{mm}$$

This pressure represents the driving force required to extrude the material through the nozzle.

### 2.4. Dimensional Accuracy

Dimensional accuracy was assessed according to Liu et al. [29]. The printed cube dimensions (length, width, height) were measured with a caliper, and dimensional deviation (%) was calculated using:

Deviation (%) = (Measured value − Target value)/Target value × 100

A positive deviation indicates a printed pattern that is thicker than that designed, whereas a negative deviation denotes a thinner pattern.

### 2.5. Quality Characteristics

#### 2.5.1. Moisture Content

The moisture content of the samples was determined by the direct gravimetric method [30] using a drying oven (Memmert UF110, Schwabach, Germany). Approximately 5 g of each homogenized sample was accurately weighed into pre-dried and pre-weighed aluminum dishes. The dishes were placed in a ventilated oven at 130 °C and left to dry overnight. Drying continued until a constant weight was reached, defined as a difference of less than 0.005 g between two successive weighings. The moisture content was expressed as a percentage of the initial sample weight.

#### 2.5.2. Colorimetry

The color of the experimental samples was evaluated using a calibrated colorimeter (Elscolab—Hunterlab—ColorFlex EZ with Glass Sample Cup 64 mm). Measurements were performed in the CIE Lab* color space, where L* represents lightness, a* represents the red-green axis and b* represents the yellow-blue axis. The colorimeter was standardized using a black and white reference tile before each session.

#### 2.5.3. Protein Content

The protein content of the samples was determined using the Kjeldahl method [30]. Approximately 1 g of homogenized sample was digested in concentrated sulfuric acid in the presence of a copper catalyst using a FOSS Digestor 2020 (FOSS, Hillerød, Denmark) until complete mineralization was achieved, resulting in the conversion of organic nitrogen to ammonium sulfate. The digest was then neutralized with sodium hydroxide, and the liberated ammonia was distilled into a boric acid solution using a FOSS Kjeltec 2200 distillation unit (FOSS, Denmark). The distillate was subsequently titrated with standardized hydrochloric acid to quantify the nitrogen content. The crude protein content was calculated by multiplying the total nitrogen content by a conversion factor of 5.7, as recommended for plant-based proteins [31].

### 2.6. IDDSI Measurements

Textural properties of solid and semi-solid foods were assessed according to the International Dysphagia Diet Standardisation Initiative. Each of the eight levels is associated with standardized texture classification and reproducible test methods. Assessments were conducted using the spoon tilt, fork drip and fork pressure tests described in the IDDSI framework [12]. The spoon tilt test involves tilting a standard spoon containing the sample to observe its ability to slide or retain shape, whereas the fork drip test measures the extent to which a sample holds between the fork prongs or flows through them. The fork pressure test is performed by applying slight pressure with the thumb to the fork until blanching of the thumbnail is observed, thereby providing an index of sample softness and compressibility. All experimental samples were evaluated using these procedures to determine their classification within the framework [20].

### 2.7. Texture Profile Analysis

The Texture Profile Analysis (TPA) is a widely used imitative test in food technology that simulates the mechanical conditions experienced by foods in the oral cavity during mastication allowing rapid and reproducible assessment of multiple textural attributes [32]. TPA was performed using a texturometer (Lloyd TA1, Ametek, Berwyn, PA, USA) equipped with a 50 mm cylindrical probe and subsequently measured the hardness, cohesiveness, adhesiveness (negative area), gumminess, springiness and chewiness by a two-bite compression test at room temperature. The same cubic samples (25 × 25 × 25 mm) used for dimensional accuracy assessment were analyzed. TPA measurements were conducted in compression mode at 30% strain relative to the original sample height, applied uniformly to the entire structure, with a pretest speed of 10 mm/min, a test speed of 0.8 mm/s and a waiting time of 1.5 s, using a trigger force of 0.08 N.

### 2.8. Exploratory Sensory Evaluation

A total of 17 healthy adult participants (7 females and 10 males aged between 25 and 40 years old) took part in the exploratory sensory assessment conducted in the Laboratory of Gastronomic Sciences (Gembloux Agro-Bio Tech, University of Liège, Gembloux, Belgium). All participants were untrained (no knowledge of IDDSI classification) and provided written informed consent prior to testing. According to local regulations, this sensory study did not require formal ethical approval.

Four samples (reference and three TMFs) were presented in randomized order and evaluated independently. Participants scored the texture, the taste appreciation and the visual appreciation using six descriptors (hardness, cohesiveness, gumminess, grittiness, melting texture and moisture) on a five-point scale (from “completely disagree” to “completely agree”). For statistical analysis, the five-point Likert responses were converted into numerical scores ranging from 1 to 5 respectively.

### 2.9. Statistical Analysis

All statistical analyses were conducted to assess differences among the formulations and ensure the robustness of the reported results. Unless otherwise specified, all measurements were performed in triplicate (*n* = 3), corresponding to samples from the same batch, and are expressed as mean ± standard deviation (SD). Data processing was carried out using GraphPad Prism (version 8.0.1, GraphPad Software Corporation, San Diego, CA, USA), Microsoft Excel (Excel 2021, Microsoft Corporation, Redmond, WA, USA) and RStudio (version 2023.06.1 +524, Posit Software, Boston, MA, USA). Prior to selecting the appropriate statistical tests, data distribution and variance homogeneity were examined through Shapiro–Wilk normality tests, QQ plots and homoscedasticity assessments. When the data met parametric assumptions, one-way ANOVA followed by multiple comparison procedures was applied to identify significant differences between formulations. In contrast, when normality or homoscedasticity criteria were not satisfied, non-parametric analyses were performed using the Kruskal–Wallis test followed by Dunn’s post hoc test. Statistical significance was consistently set at *p* < 0.05.

## 3. Results

### 3.1. Extrusion Behavior

**Figure 1:**
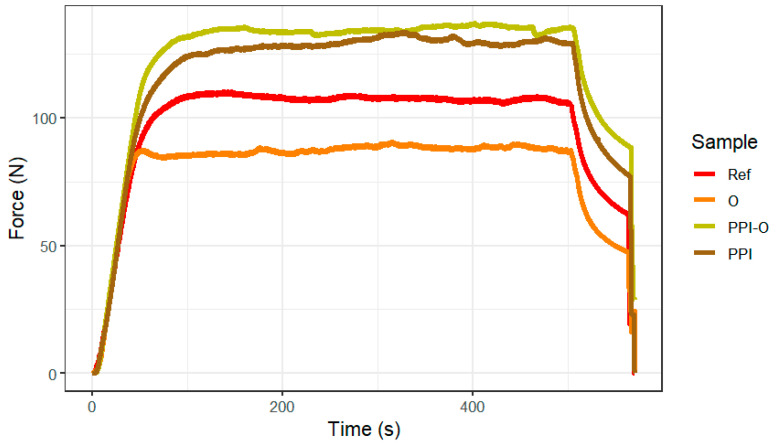
Extrusion force profiles obtained during compression of formulations using a texture analyzer. REF, reference. O, formulation with only oleogel. PPI-O, formulation with pea protein isolate and oleogel. PPI, formulation with only pea protein isolate. Each curve corresponds to the mean profile of triplicate measurements (*n *= 3).

Extrusion force profiles during 3D printing showed clear formulation-dependent differences as shown by Figure 1. All samples presented a rapid increase in force followed by a stable plateau characteristic of continuous extrusion. As summarized in Table 2, the oleogel formulation (O) exhibited the lowest steady-state force (87.69 N) and the lowest compression energy (2398 N·mm), indicating easier flow and reduced resistance during syringe extrusion. The reference formulation (Ref) showed intermediate extrusion behavior with a plateau force of 107.65 N and 2911 N·mm of compression energy. Formulations containing pea protein isolate (PPI-O and PPI) demonstrated the highest resistance to compression. PPI-O and PPI showed elevated steady-state forces (134.43 N and 129.06 N) and the highest energy requirements (3664 N·mm and 3476 N·mm, respectively). Extrusion pressure ranged from 124.05 kPa (O) to 190.16 kPa (PPI-O).

### 3.2. Dimensional Accuracy

**Figure 2:**
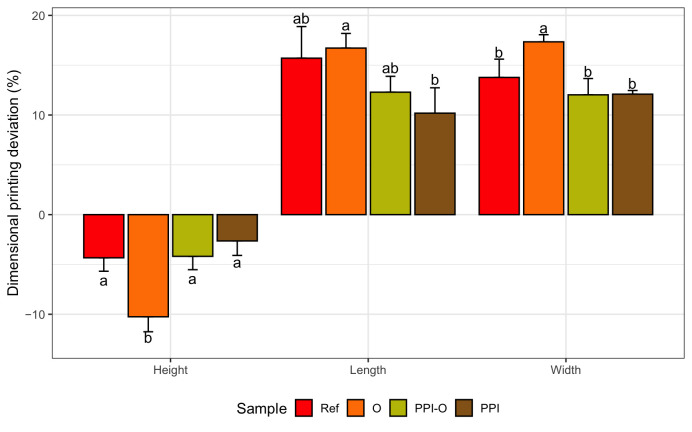
Dimensional printing deviation of 3D-printed cubes. Ref, reference. O, formulation with only oleogel. PPI-O, formulation with pea protein isolate and oleogel. PPI, formulation with only pea protein isolate. Data are expressed as mean ± standard deviation (*n *= 3). Data were analyzed using a one-way ANOVA with Tukey’s post hoc test (*p* < 0.05). Different letters in the same column indicate a significant difference.

**Figure 3:**
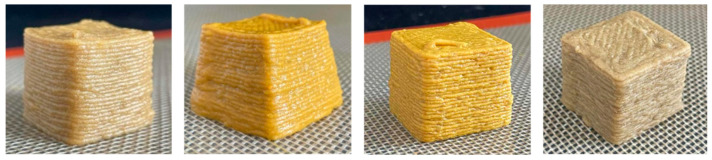
Side view of the 3D-printed cubes to illustrate dimensional printing deviations. Left to right: Ref, reference. O, formulation with only oleogel. PPI-O, formulation with pea protein isolate and oleogel. PPI, formulation with only pea protein isolate.

Concerning the printing stability, a dimensional printing deviation study was conducted. Figure 2 shows the dimensional printing deviations of the different 3D-printed formulations based on the dimensions in Table 3 compared to the designed model. The oleogel-only sample (O) presented the largest significant deviations in all three dimensions, particularly in height (22.44 ± 0.38 mm compared to 25.0 mm) and width (29.18 ± 0.37 mm compared to 25.0 mm), indicating poor structural stability during layer deposition. In contrast, the reference formulation (Ref) and those containing pea protein (PPI and PPI-O) demonstrated significantly improved fidelity to the designed model, with reduced deviations and more consistent shapes. These observations are illustrated in Figure 3, which presents the printed cubes for visual comparison.

### 3.3. Quality Characteristics

The quality characteristics of TMFs for dysphagia are summarized in Table 4. The moisture content decreased with the addition of ingredients such as PPI or oleogel, with values ranging from 73.25 ± 0.42% (Ref) to 66.09 ± 0.11% (PPI-O). All samples nevertheless showed high moisture levels.

Regarding the color parameters, lightness (L*) values ranged from 48.40 ± 0.14 (PPI) to 53.58 ± 0.00 (O). The redness (a*) values were highest in the O sample (12.96 ± 0.00) and lowest in the PPI sample (7.82 ± 0.01). The yellowness (b*) values also differed between formulations, with the O sample showing the highest value (66.49 ± 0.05), followed by PPI-O (62.63 ± 0.06). The Ref and PPI samples exhibited lower b* values (29.79 ± 0.03 and 28.46 ± 0.03, respectively). We can observe that PPI-O and O resemble each other because of the presence of curcumin (ΔE ≈ 33–37), while the Reference and PPI cluster together without curcumin (ΔE = 1.4), indicating that the intrinsic color of the lentils becomes the dominant chromatic factor.

Concerning protein content, the addition of pea protein isolates in formulations PPI-O and PPI resulted in significantly higher crude protein levels, reaching 8.62 ± 0.21% and 9.35 ± 0.20%, respectively, compared with 6.07 ± 0.41% in the reference sample.

### 3.4. IDDSI Measurements

The IDDSI test results for TMFs are shown in Table 5. All TMFs exhibited sufficient cohesiveness and structural integrity to maintain their shape on the spoon. When the spoon was tilted sideways, the samples slipped off with a slight shake, leaving only a small residue. In the fork drip test, all TMFs were retained on the fork without flowing or dripping through the tines. During the fork press test, the samples were easily deformed and passed through the fork slots, producing a distinct pattern on the surface. The material could be readily mashed with minimal pressure and did not regain its shape after compression [12].

Although the spoon tilt, fork drip, and fork pressure tests showed cohesive behavior similar to Level 4 foods, the presence of visible lentil fragments resulted in classification as Level 5 (Minced & Moist) according to the IDDSI particle size criterion. Based on visual observation, no particles appeared to exceed the 4 mm size criterion associated with Level 5 [12,33]. Because the IDDSI tests are primarily classification tools and may not fully distinguish subtle differences between formulations, additional instrumental analyses such as Texture Profile Analysis (TPA) are recommended to further characterize the samples.

### 3.5. Texture Profile Analysis

**Figure 4:**
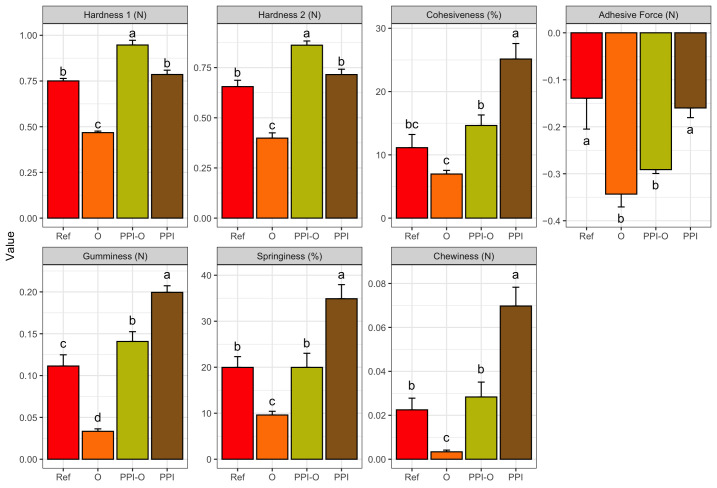
Hardness bite 1, hardness bite 2, cohesiveness, adhesive force, gumminess, springiness and chewiness according to TMF formulations. Ref, reference. O, formulation with only oleogel. PPI-O, formulation with pea protein isolate and oleogel. PPI, formulation with only pea protein isolate. Data are expressed as mean ± standard deviation (*n *= 3). Data were analyzed using a one-way ANOVA with Tukey’s post hoc test (*p* < 0.05). Different letters in the same column indicate a significant difference.

The Texture Profile Analysis (TPA) results are presented in Figure 4. Significant differences were observed among formulations for all mechanical parameters. For hardness (first and second bite), the PPI-O formulation exhibited the highest significant values, followed by PPI and the Ref formulations, whereas the oleogel-only (O) formulation showed the lowest values. Cohesiveness and gumminess followed a similar trend, with the PPI formulation presenting the highest significant values and O formulation the lowest. The PPI-O and Ref formulations consistently displayed intermediate values between these two samples. All four formulations were significantly different from one another, although PPI-O was statistically closer to the reference than the other TMF formulations. For springiness and chewiness, the O formulation again presented the lowest significant values and the PPI formulation the highest significant. The reference and PPI-O formulations were not significantly different for these parameters. Adhesive force also differed among formulations. The reference and PPI samples showed similar values, whereas the O and PPI-O formulations exhibited higher adhesive values. Overall, the incorporation of PPI increased mechanical strength across all TPA parameters, whereas the oleogel-only formulation consistently exhibited the weakest mechanical performance. This agrees with the dimensional printing deviation results, where the printed constructions of this sample tended to lose shape after 3D printing, confirming that the oleogel alone does not provide sufficient structural support. The hybrid PPI-O formulation showed intermediate behavior, frequently approaching the values of the reference sample.

### 3.6. Exploratory Sensory Evaluation

**Figure 5:**
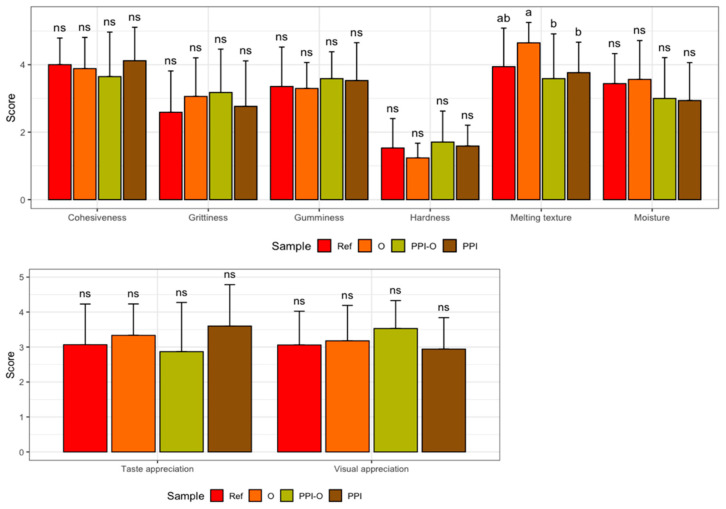
Exploratory sensory evaluation results. Ref, reference. O, formulation with only oleogel. PPI-O, formulation with pea protein isolate and oleogel. PPI, formulation with only pea protein isolate. Data are expressed as mean ± standard deviation (*n* = 17). Data were analyzed using Kruskal–Wallis test followed by Dunn’s post hoc test (*p* < 0.05). Different letters indicate a significant difference. ns, no significant difference.

Exploratory sensory evaluation results are presented in Figure 5. No significant differences were observed among most formulations for cohesiveness, grittiness, gumminess, hardness, or moisture. However, the oleogel-only (O) sample exhibited a significantly higher melting texture compared with the PPI-containing formulation, indicating that this attribute was the only discriminant factor detected by the panel. Although non-significant, visual inspection of the data revealed minor trends, including slightly higher hardness and gumminess scores for the PPI and PPI-oleogel samples and lower hardness for the oleogel-only sample. Cohesiveness scores remained relatively uniform across formulations. Taken together, all products received mid-range scores for both visual and taste-based appreciation, and the sensory panel perceived the samples as generally soft, moderately moist and cohesive. These results suggest that the formulations were largely similar from a sensory standpoint, with melting texture being the only attribute showing a statistically detectable difference.

## 4. Discussion

### 4.1. Principal Findings

This study provides an integrated evaluation of the printability, structural integrity, compositional content and multimodal textural behavior of 3D-printed TMFs formulated with pea protein isolate, a curcumin-enriched oleogel, or their combination. To our knowledge, the combined incorporation of pea protein isolate and curcumin-enriched oleogel within a single 3D-printed texture-modified food formulation has not been previously reported for dysphagia-oriented applications.

Across all analyses, the formulations exhibited distinct techno-functional behaviors: PPI consistently reinforced the matrix, improving mechanical strength and dimensional stability, whereas the oleogel produced softer, less elastic and less cohesive structures while enabling the incorporation of lipophilic bioactive compounds. The hybrid PPI–O formulation exhibited intermediate properties, combining suitable printability with good shape fidelity and balanced texture while providing higher protein content than the reference formulation. While all samples were classified as compatible with IDDSI Level 5, this formulation provided the most favorable compromise for developing visually appealing 3D-printed TMFs.

### 4.2. Comparison with Prior Work

Regarding printability and structural behavior, the results were consistent with previous findings reporting that plant proteins enhance network formation and water binding in semi-solid matrices [10,20]. Accordingly, the PPI-containing samples (PPI and PPI-O) required higher extrusion forces and showed improved dimensional accuracy after 3D printing, reflecting the formation of a stronger protein-based network that supports structural stability during layer-by-layer deposition [17,21]. In contrast, the oleogel-only formulation showed lower extrusion resistance and greater dimensional deviation, consistent with the lubricating and structure-softening effects of oleogels reported in semi-solid food systems [24,25].

Nevertheless, the measured X and Y dimensions of the printed cubes were slightly larger than the target geometry (25 × 25 × 25 mm). Such deviations are common in extrusion-based food printing and mainly result from viscoelastic relaxation at the nozzle exit (die swell) and lateral spreading of the deposited filament. In semi-solid food materials, the extruded strand may expand and flatten upon deposition, increasing the effective line width and consequently the overall printed dimensions. Similar phenomena have been reported in previous studies [29,34,35]. Because all formulations were printed under identical processing conditions, this systematic deviation does not affect the comparative evaluation of dimensional stability among samples.

Quality characteristics further support these formulation-dependent behaviors. The decrease in moisture content following PPI or oleogel addition is consistent with the high water-binding capacity of proteins [10] and reports that oleogel structuring can influence water mobility [26]. Despite this reduction, all samples retained relatively high moisture contents (66–73%), which may facilitate bolus formation and swallowing. Higher initial food moisture reduces the need for saliva incorporation during oral processing, an important consideration for individuals with dysphagia [36], who frequently experience dehydration and reduced salivary flow.

Although the addition of pea protein isolate significantly increased protein content, the present study did not evaluate protein digestibility or amino acid availability, and therefore no conclusions can be drawn regarding nutritional quality. Beyond its techno-functional role, PPI is a plant-based, low-allergen protein source suitable for elderly or medically fragile populations and aligns with sustainability priorities due to the lower environmental impact of plant proteins [22,37].

The higher b^*^ colorimetric values observed in oleogel-containing samples reflect curcumin pigmentation, consistent with reports describing enhanced chromatic intensity when curcumin is incorporated into lipid carriers [23,27]. ΔE-based color analysis confirmed that oleogel-containing samples formed a distinct chromatic cluster, whereas the Ref and PPI formulations remained closely aligned. Despite significant instrumental differences, sensory evaluation indicated that participants did not perceive notable color differences during texture assessment. However, this result should be interpreted cautiously, as visual appearance is a primary driver of appetite in elderly populations with reduced olfactory and gustatory sensitivity. Consequently, color differences may play a more critical role if the same formulations are assessed by individuals with dysphagia [8,9].

Although 3D food printing is often promoted for improving visual appeal through shape customization, this study intentionally employed a simple cubic geometry to minimize confounding variables and focus on formulation-driven textural and sensory responses. Consequently, the esthetic potential of 3D printing was not directly evaluated and should be explored in future studies.

Texture characterization using IDDSI tests, TPA and sensory analysis showed strong internal consistency. All formulations were classified as compatible with IDDSI Level 5. As reported in prior work, the IDDSI framework is mainly intended for safety classification, but does not constitute clinical validation of swallowing safety or patient-specific suitability and is less sensitive to subtle formulation-dependent differences [32,33]. This limited discriminatory power was evident here, as all samples were categorized identically despite structural and mechanical differences identified through instrumental measurements.

TPA results provided a more detailed differentiation of textural properties. The oleogel-only formulation exhibited very low cohesiveness combined with high adhesiveness, which may increase bolus fragmentation and residue formation despite its low hardness [36]. In contrast, the PPI-only formulation showed high firmness, gumminess, and chewiness, potentially increasing oral processing effort and limiting suitability for individuals with reduced masticatory capacity. This trend also aligns with observations from Wee et al. (2018), who reported that these parameters reflect their more elastic and internally bonded matrices and correlate strongly with oral processing behavior and structural density [38]. The hybrid PPI-O formulation exhibited increased cohesiveness and reduced adhesiveness while maintaining moderate chewiness. This balance is advantageous for dysphagic individuals, as it promotes bolus integrity, facilitates controlled oral transport, and reduces the likelihood of oral and pharyngeal residue [36]. These properties are also consistent with synergistic protein-lipid structuring effects reported previously [10,39].

Exploratory sensory evaluation supported these instrumental findings. While untrained assessors detected few significant differences, melting texture was rated significantly higher for the oleogel-only sample, consistent with its lower instrumental texture parameters, confirming close agreement between sensory perception and mechanical measurements. A melt-in-the-mouth perception may reduce mastication demands and facilitate bolus formation, but excessive melting without sufficient cohesion may impair bolus control, highlighting the balance achieved by the PPI–O formulation.

The limited ability of the untrained panel to detect clear differences likely reflects the small number of assessors and the insufficient sensitivity and standardization of the sensory protocol, a challenge previously described in sensory testing of dysphagia-oriented matrices [3]. Therefore, the fact that IDDSI could not differentiate samples and that the panel only distinguished melting behavior underscores the importance of combining classification tools, refined sensory methodologies, and instrumental methods for comprehensive characterization of TMFs.

Xanthan gum is widely recognized as a key thickening agent in dysphagia management [32] and likely played a central structuring role across all formulations. Its pronounced shear-thinning behavior facilitates extrusion and enables rapid structural recovery after deposition [7,18]. Its influence on hardness, adhesiveness and cohesiveness [40] may have contributed to the dimensional outcomes observed and to the similarity in IDDSI classification.

Unlike several previous studies, this work used a pharmacy-grade dysphagia thickener as a reference rather than a conventional non-dysphagic food product. This reference formulation consisted of a multi-component thickening system, including dextrin-maltose, xanthan gum, carrageenan, and erythritol, whereas the experimental formulations were structured exclusively with xanthan gum. The reference therefore served as a practical benchmark representative of products currently prescribed in dysphagia management rather than a formulation-matched control.

### 4.3. Limitations

This study presents several limitations. First, the forces measured during the TPA were very small, and no universal reference thresholds exist for dysphagia-oriented foods. Because TPA results depend strongly on matrix composition and testing parameters, slight variations arising from sample preparation or storage further reduce the interpretability of absolute values. For these reasons, they should be interpreted as relative indicators for comparing formulations.

Another limitation of this study is the absence of fundamental rheological characterization (e.g., shear viscosity or oscillatory measurements). Such analyses would provide deeper insight into flow behavior, yield stress, and structural recovery, and would help better predict printability and post-deposition stability.

Moreover, although the oleogel enabled the incorporation of curcumin into the printed matrix, its chemical stability after extrusion and storage, bioaccessibility and functional impact were not evaluated. Consequently, curcumin served as a model lipophilic compound rather than as a validated functional ingredient. In the present work, references to bioactive compound delivery through oleogel-based systems were limited to previously published studies [41,42] and should be interpreted as literature-based context rather than effects demonstrated in this study.

Furthermore, as already mentioned, although PPI increased protein content, protein quality parameters such as digestibility and amino acid availability were not assessed, limiting conclusions regarding nutritional benefit.

Additionally, all analyses were conducted shortly after printing, and the effects of storage time, temperature, or reheating on texture, moisture distribution, and structural stability were not investigated. These factors are highly relevant for real-world implementation in clinical and care settings.

Finally, sensory evaluation was conducted only with non-dysphagic individuals, limiting the applicability to the target dysphagic population. As the study is exploratory in nature and involved a relatively small panel, the ability to detect subtle sensory differences between formulations may also be limited. Moreover, this work did not fully explore the role of visually driven acceptability, as only simple cube geometries were printed for standardization.

## 5. Conclusions

This study demonstrates the potential of extrusion-based 3D food printing to design TMFs tailored to the specific needs of individuals with dysphagia through rational formulation strategies. By combining PPI with a curcumin-enriched oleogel, it was possible to obtain a plant-based TMF exhibiting adequate printability, enhanced protein composition, and a balanced textural profile compatible with IDDSI texture requirements. These findings highlight the relevance of 3D printing as a flexible platform for the development of next-generation dysphagia-oriented foods and support further investigations involving clinical validation, nutritional quality assessment, and patient-centered design.

### Future Directions

Although 3D food printing shows promise for dysphagia management, several challenges still limit clinical adoption, including safety considerations, equipment costs and the technical expertise required in healthcare settings. The limited involvement of dysphagic patients in sensory and clinical evaluations also restricts understanding of real-world acceptability and swallowing safety. Further work should focus on advanced rheological characterization, digestion studies to assess bioactive stability and bioavailability, and optimization of printing parameters and post-processing conditions. In addition, co-design approaches involving clinicians, caregivers and patients could support the development of nutritionally tailored and clinically acceptable 3D-printed foods [16].

## Figures and Tables

**Table 1:** Ingredients used and proportions (expressed in weight percentage) of the different texture-modified food formulations for dysphagia. LC Base, Lentil-Carrot Base. XG, Xanthan Gum. PPI, Pea Protein Isolate. Ref, reference. O, formulation with only oleogel. PPI-O, formulation with Pea Protein Isolate and oleogel. PPI, formulation with only Pea Protein Isolate.

Samples	LC Base (%)	Instant Thickener (%)	XG (%)	PPI (%)	Oleogel (%)
Ref	96	4	–	–	–
O	91.6	–	0.4	–	8
PPI-O	87.1	–	0.4	4.5	8
PPI	95.1	–	0.4	4.5	–

**Table 2:** Mechanical parameters quantifying extrusion behavior of the formulations. Ref, reference. O, formulation with only oleogel. PPI-O, formulation with pea protein isolate and oleogel. PPI, formulation with only pea protein isolate. Data are expressed as mean ± standard deviation (*n *= 3). Data were analyzed using a one-way ANOVA with Tukey’s post hoc test (*p* < 0.05). Different letters in the same column indicate a significant difference.

Samples	Steady-State Force (N)	Compression Energy (N·mm)	Extrusion Pressure (kPa)
Ref	107.65 ± 6.23 ^b^	2911 ± 101 ^b^	152.29 ± 8.82 ^b^
O	87.69 ± 2.45 ^c^	2398 ± 57 ^c^	124.05 ± 3.46 ^c^
PPI-O	134.43 ± 3.21 ^a^	3664 ± 74 ^a^	190.16 ± 4.54 ^a^
PPI	129.06 ± 10.05 ^a^	3476 ± 251 ^a^	182.57 ± 14.23 ^a^

**Table 3:** 3D-printed cubes’ dimensions. Ref, reference. O, formulation with only oleogel. PPI-O, formulation with pea protein isolate and oleogel. PPI, formulation with only pea protein isolate. Data are expressed as mean ± standard deviation (*n *= 3). Data were analyzed using a one-way ANOVA with Tukey’s post hoc test (*p* < 0.05). Different letters in the same column indicate a significant difference.

Samples	Height (mm)	Length (mm)	Width (mm)
Ref	23.92 ± 0.34 ^a^	28.44 ± 0.46 ^b^	29.93 ± 0.79 ^ab^
O	22.44 ± 0.38 ^b^	29.34 ± 0.18 ^a^	29.18 ± 0.37 ^a^
PPI-O	23.95 ± 0.33 ^a^	28.01 ± 0.41 ^b^	28.07 ± 0.40 ^ab^
PPI	24.34 ± 0.36 ^a^	28.02 ± 0.09 ^b^	27.55 ± 0.64 ^b^

**Table 4:** Quality characteristics of texture-modified food formulations. Ref, reference. O, formulation with only oleogel. PPI-O, formulation with pea protein isolate and oleogel. PPI, formulation with only pea protein isolate. Data are expressed as mean ± standard deviation (*n *= 3). Data were analyzed using a one-way ANOVA with Tukey’s post hoc test (*p* < 0.05). Different letters in the same column indicate a significant difference.

Samples	Moisture Content (%)	Crude Protein (%)	Color Values			
			L*	a*	b*	∆E
Ref	73.25 ± 0.42 ^a^	6.07 ± 0.41 ^c^	48.82 ± 0.02 ^c^	8.11 ± 0.00 ^c^	29.79 ± 0.03 ^c^	
O	68.79 ± 0.14 ^c^	6.22 ± 0.13 ^c^	53.58 ± 0.00 ^a^	12.96 ± 0.00 ^a^	66.49 ± 0.05 ^a^	37.32 ± 0.05
PPI-O	66.09 ± 0.11 ^d^	8.62 ± 0.21 ^b^	51.94 ± 0.00 ^b^	12.34 ± 0.00 ^b^	62.63 ± 0.06 ^b^	33.25 ± 0.06
PPI	71.75 ± 0.07 ^b^	9.35 ± 0.20 ^a^	48.40 ± 0.14 ^d^	7.82 ± 0.01 ^d^	28.46 ± 0.03 ^d^	1.42 ± 0.06

**Table 5:** Categorization of texture-modified food formulations using International Dysphagia Diet Standardisation Initiative evaluation methods. Ref, reference. O, formulation with only oleogel. PPI-O, formulation with pea protein isolate and oleogel. PPI, formulation with only pea protein isolate. Results correspond to three independent evaluations. International Dysphagia Diet Standardisation Initiative 2019 [12].

Samples	IDDSI Test			
	Spoon Tilt Test	Fork Drip Test	Fork Pressure Test	Level
Ref	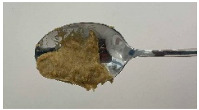	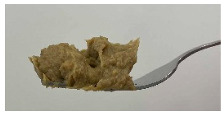	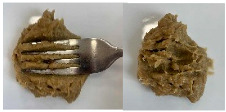	Level 5
O	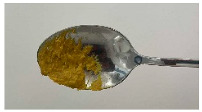	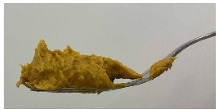	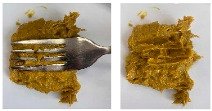	Level 5
PPI-O	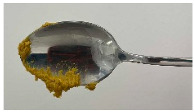	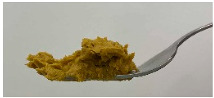	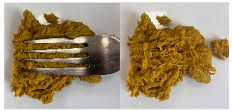	Level 5
PPI	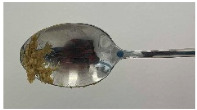	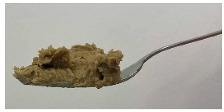	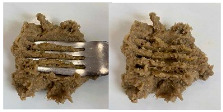	Level 5

## Data Availability

The data presented in this study are available on request from the corresponding authors due to privacy considerations.

## Abbreviations

The following abbreviations are used in this manuscript:

TMFs	Texture-modified foods
3D	3-Dimensional
O	Oleogel
PPI	Pea Protein Isolate
IDDSI	International Dysphagia Diet Standardisation Initiative
TPA	Texture Profile Analysis
